# lncRNA DCST1-AS1 Facilitates Oral Squamous Cell Carcinoma by Promoting M2 Macrophage Polarization through Activating NF-*κ*B Signaling

**DOI:** 10.1155/2021/5524231

**Published:** 2021-08-08

**Authors:** Yilong Ai, Shiwei Liu, Hailing Luo, Siyuan Wu, Haigang Wei, Zhe Tang, Xia Li, Chen Zou

**Affiliations:** ^1^Foshan Stomatological Hospital, School of Stomatology and Medicine, Foshan University, Foshan 528000, China; ^2^Department of Stomatology, Foshan First People's Hospital, Foshan 528000, China

## Abstract

lncRNAs are related to the progression of various diseases, including oral squamous cell carcinoma (OSCC), which is a common squamous cell carcinoma of the head and neck. Tumor-associated macrophages and tumor cells are significant components of tumor microenvironment. M2 polarization of tumor-associated macrophages is a crucial actor in tumor malignancy and metastasis. In this study, we studied the molecular mechanism of lncRNA DCST1-AS1 in OSCC. Here, we reported that DCST1-AS1 was significantly increased in OSCC cells. We found that loss of DCST1-AS1 obviously inhibited the proliferation, migration, and invasion of OSCC cells and xenograft tumor growth. Meanwhile, silencing of DCST1-AS1 also repressed the percentage of macrophages expressing M2 markers CD206 and CD11b. DCST1-AS1 shRNA enhanced the percentage of macrophages expressing M1 markers CD80 and CD11c. Then, we observed that loss of DCST1-AS1 suppressed OSCC progression via inactivating NF-*κ*B signaling. As well established, NF-*κ*B signaling exerts critical roles in tumor progression, and our study proved that DCST1-AS1 could regulate NF-*κ*B signaling. We proved that blocking the NF-*κ*B pathway using antagonists greatly downregulated OSCC progression and M2 macrophage polarization induced by the overexpression of DCST1-AS1. To sum up, we reported that DCST1-AS1 plays an important role in modulating OSCC tumorigenicity and M2 macrophage polarization through regulating the NF-*κ*B pathway.

## 1. Introduction

Head and neck cancer is a sixth most frequent malignant tumor worldwide [1]. OSCC is the most common type of head and neck cancer [2]. Almost 300000 new cases are reported each year, and over 140000 patients die due to OSCC [3]. Currently, surgery, radiation, and chemoradiation are the primary treatment for OSCC. Great progress is made, but the overall 5-year survival of OSCC patients still remained low [4]. Hence, the molecular mechanisms of OSCC progression and development are in urgent need to identify more treatment.

Macrophages are significant players in both the innate and adaptive immune systems. Recently, macrophages are classified as M1 macrophages with proinflammatory activity and M2 macrophages with anti-inflammatory effects [5]. In addition, growing evidence has reported that M2 macrophages accelerate tumor progression and metastasis [6]. Tumor-associated macrophages are termed as a macrophage population educated by cancer cells. They exert important roles in tumor microenvironment [7]. The accelerating effect of M2 macrophages on tumor progression is regulated through complex cross-talk mechanisms. However, the underlying mechanisms by which OSCC reeducate these macrophages to elicit this polarization program remain uninvestigated.

lncRNAs is noncoding RNA with a length over 200 nt [8–10]. lncRNAs can modulate the expression of genes involving multiple physiological and pathological processes [11–13]. Current studies report that abnormal lncRNAs are closely associated with many diseases, including tumors [14, 15]. For example, lncRNA-RMRP can promote bladder cancer progression through miR-206 [16]. lncRNA-ATB can promote apoptosis of lung cancer cells by regulating miR-200a [17]. In addition, lncRNA B3GALT5-AS1 represses colon cancer metastasis through sponging miR-203 [18].

Moreover, lncRNAs are shown to be related to the progression of OSCC. For instance, HOXA11-AS can promote OSCC development through sponging miR-98-5p and upregulating YBX2 [19]. Silencing of lncRNA LEF1-AS1 represses OSCC progression via modulating Hippo signaling [20]. Additionally, lncRNA SNHG5 can induce OSCC cell growth by sponging miR-655-3p and regulating FZD4 [21]. lncRNA DCST1-AS1 has been reported in many cancers. DCST1-AS1 induces the TGF-*β*-induced EMT process and chemoresistance in breast cancer [22]. lncRNA DCST1-AS1 can contribute to hepatocellular carcinoma progression via regulating AKT/mTOR signaling [23]. However, the function and detailed mechanism of DCST1-AS1 in OSCC progression are barely known.

In our present study, we found that DCST1-AS1 was aberrantly increased in OSCC and contributed to OSCC growth in vitro and in vivo. In addition, silencing of DCST1-AS1 significantly inhibited M2-like polarization of macrophages. In addition, DCST1-AS1 induced OSCC development by enhancing the NF-*κ*B signaling pathway.

## 2. Methods and Materials

### 2.1. Cell Culture

A normal human oral epithelial cell line (NHOK) and oral squamous cell lines (SCC-9, FADU, Cal-27, SCC-25, and HN4) were acquired from the American Type Culture Collection (ATCC, Rockville, MD, USA). Cells were incubated in DEME (Invitrogen, Carlsbad, CA, USA) medium added with 10% FBS with 5% CO_2_ at 37°C.

### 2.2. Cell Transfection

The pcDNA-DCST1-AS1 overexpression vector and the NC vector were constructed by GenePharma (Shanghai, China). A shRNA plasmid against lncRNA DCST1-AS1 (sh-DCST1-AS1) or sh-NC was ordered from GenePharma (Shanghai, China). A Lipofectamine 3000 transfection reagent (Invitrogen, USA) was used based on the manufacturer's instruction.

### 2.3. CCK-8 Assay

Cells were grown into 96-well plates added with 200 *μ*L DMEM culture medium (2 × 10^3^ cells per well). Then, the CCK-8 kit (TransDetect, China) was used to assess cell viability at days 1, 2, and 3, respectively, after treatment. Absorbance was tested at 570 nm on a microplate reader (Bio-Rad, USA).

### 2.4. EdU Assay

After transfection, cells (1 × 10^4^) were grown into 96-well plates each well. The EdU assay kit (RiboBio, China) was utilized to detect cell proliferation. To obtain the images, a fluorescence microscope (Nikon, Japan) was used.

### 2.5. Transwell Assay

Cell migration and cell invasion were evaluated using 24-well Biocoat cell culture inserts (Corning, NY, USA) with a polyethylene terephthalate membrane (8 *μ*m pores) coated without or with the Matrigel Basement Membrane Matrix. After transfection, cells were seeded at a density of 5 × 10^4^ cells in 100 *μ*L serum-free DMEM. Cells were plated onto the upper chambers. Lower chambers were added using 600 *μ*L medium with 20% FBS. 48 hours later, the membranes were fixed with 4% methanol and stained with 0.1% crystal violet.

### 2.6. RNA Sequencing and Analysis

Total RNA was extracted using the TRIzol reagent (Invitrogen, Carlsbad, CA, USA) according to standard protocols. Cell RNA-seq (3 replicates each group) was carried out in RiboBio (Guangzhou, China). Briefly, intact RNA was fragmented, end repaired, adapter ligated, and PCR amplified. Libraries were sequenced by Illumina HiSeq 2000 (Illumina, San Diego, USA). The sequenced reads were aligned to the human reference genome (H19) using TopHat v1.4.1. Differential gene expression (DEG) analysis was done with DEseq and DEGseq (cell RNA-seq).

### 2.7. Quantitative Real-Time PCR

RNA extraction was carried out using the TRIzol reagent (Invitrogen, Carlsbad, CA, USA). cDNA was synthesized by using a PrimeScript RT reagent kit (TaKaRa, Japan). Quantitative detection was performed on the ABI StepOne Real-Time PCR System (San Diego, CA, USA). The primers were synthesized by Sangon Biotech (Shanghai, China) and are shown in Table 1. Gene expression was calculated based on the 2^−ΔΔCT^ method.

### 2.8. Western Blotting Analysis

In brief, 40 *μ*g protein was loaded to SDS-PAGE, and then, the PVDF membrane was used to transfer the protein. Membranes were blocked in 5% skim milk for 1 h. Antibodies against p65 (1 : 1000, Cell Signaling Technology, Beverly, USA), lamin B (1 : 1000, Cell Signaling Technology, Beverly, USA), and GAPDH (1 : 1000, Beyotime Biotechnology, Shanghai, China) were used to incubate the membranes for a whole night. Next day, the membrane was incubated with a HRP-conjugated secondary antibody for 1 h. The blots were visualized using the chemiluminescent detection kit (Pierce, Thermo Scientific, Waltham, USA).

### 2.9. Tumor Xenograft Model

SCC-9 cells (1 × 10^7^ cells) transfected with or without DCST1-AS1 shRNA were suspended in 200 *μ*L PBS and then subcutaneously injected into the flank of 6-week-old BALB/c nude female mice. After 5 weeks, the mice were sacrificed and the tumor tissues were collected for further research. The animal experiments were approved by the Animal Care and Use Committee of Foshan Stomatological Hospital, School of Stomatology and Medicine, Foshan University.

### 2.10. Immunofluorescence Assay

After culture on the microscope cover glass, cells were transfected. 48 h later, cells were fixed using 4% formaldehyde and blocked using 10% BSA for half an hour. A rabbit monoclonal anti-human CD206 and CD80 (1 : 100; Proteintech) were used to incubate the slides for a whole night at 4°C. Then, an Alexa Flour 555-conjugated anti-rabbit IgG secondary antibody (1 : 750; CST) was used. Cells were stained with DAPI (Merck, Darmstadt, Germany). A fluorescence microscope (OLYMPUS FV10-MCPSU) was used to acquire the images. ImageJ was used to quantify IHC data.

### 2.11. IHC Staining

The paraffin-embedded sections were dewaxed and rehydrated. 3% H_2_O_2_ was used to block endogenous peroxidase. Microwave heating was conducted for antigen retrieval. Then, 5% BSA was used to block nonspecific antigen at 37°C for 1 hour. The sections were incubated using a specific primary antibody against Ki-67 at 4°C. Next day, the sections were incubated with the corresponding secondary antibodies at 37°C. An Olympus light microscope was utilized to take the representative images. Image-Pro Plus was used to quantify IHC data.

### 2.12. Flow Cytometry

To assess the frequencies of CD206/CD11b and CD80/CD11c, cells were stained with anti-CD80-APC, anti-CD11c-PE, anti-CD206-FITC, or anti-CD11b-APC (BD Biosciences, San Jose, California, USA). Staining with fluorophore-conjugated secondary antibodies was carried out for flow cytometry analysis.

### 2.13. Statistical Analysis

Data were analyzed by the SPSS software program (version 19.0). Student's *t*-test was carried out for two-group comparisons. Multiple comparisons were analyzed using one-way ANOVA. *P* < 0.05 was considered to be statistically significant.

## 3. Results

### 3.1. Downregulation of DCST1-AS1 Inhibited OSCC Cell Proliferation, Migration, and Invasion In Vitro

**Figure 1:**
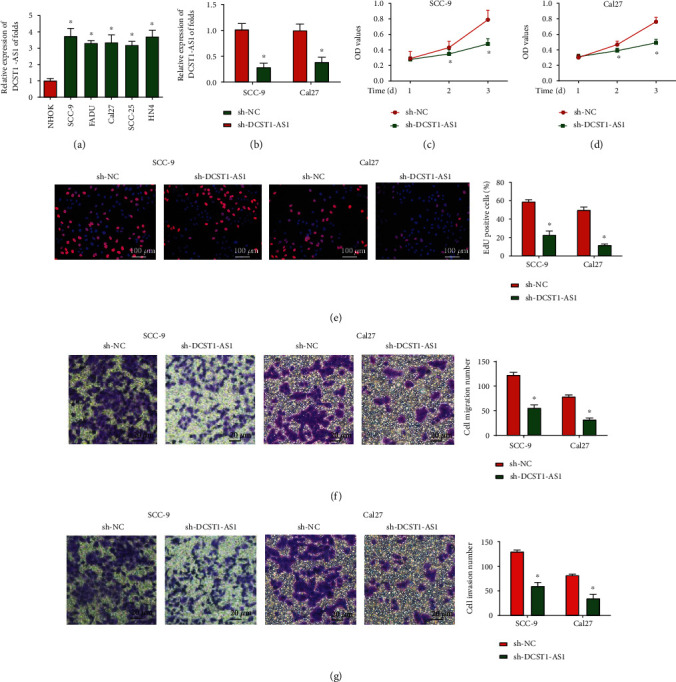
Downregulation of DCST1-AS1 reduced the progression of OSCC cells. (a) The expression of DCST1-AS1 in different types of OSCC cells (SCC-9, FaDu, Cal27, SCC-25, and HN4) and NHOK cells using qRT-PCR analysis. (b) qRT-PCR analysis of DCST1-AS1 expression in SCC-9 and Cal27 cells transfected with DCST1-AS1 shRNA or sh-NC. (c, d) CCK-8 assay was carried out to test OSCC cell viability. (e) EdU assay was performed to evaluate OSCC proliferation. (f, g) Transwell assay was utilized to test cell migration and invasion capacity. ^∗^*P* < 0.05.

The expression of DCST1-AS1 in OSCC cells was compared. We found that the expression level of DCST1-AS1 in OSCC cell lines (SCC-9, FaDu, Cal27, SCC-25, and HN4 cells) was upregulated than that in NHOK cells (Figure 1(a)). Then, SCC-9 and Cal27 cells were transfected with DCST1-AS1 shRNA or sh-NC. qRT-PCR analysis displayed that DCST1-AS1 expression was significantly decreased by DCST1-AS1 shRNA in OSCC cells as shown in Figure 1(b). Then, the CCK-8 assay was carried out, and we found that OSCC cell viability was greatly decreased by loss of DCST1-AS1 as exhibited in Figures 1(c) and 1(d). Meanwhile, the EdU assay was used to assess OSCC cell proliferation, and OSCC cell proliferation was reduced due to lack of DCST1-AS1 in Figure 1(e). In addition, we observed that OSCC cell migration and invasion capacity were obviously repressed by DCST1-AS1 shRNA (Figures 1(f) and 1(g)).

### 3.2. Decrease in DCST1-AS1 Inhibited OSCC Tumor Growth In Vivo

**Figure 2:**
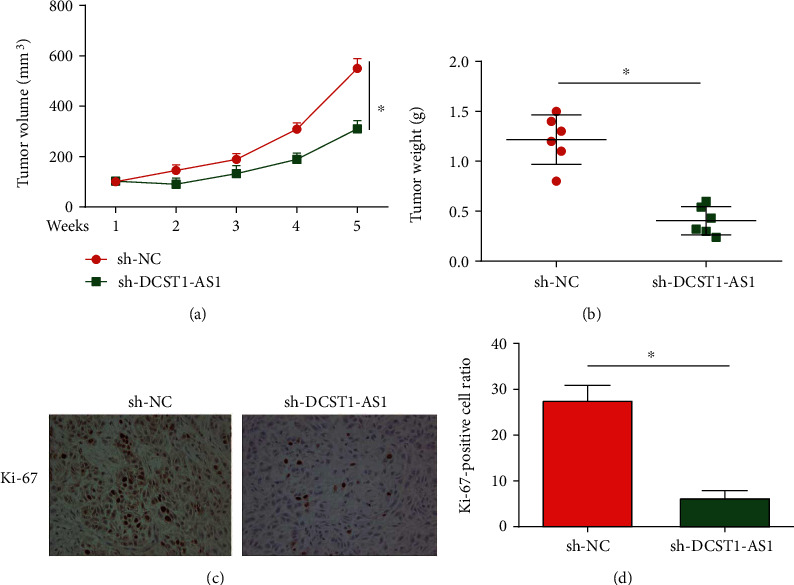
Loss of DCST1-AS1 repressed OSCC tumor growth in vivo. Dorsal flanks of nude mice were injected using 1 × 10^7^ sh-DCST1-AS1- or sh-NC-transfected SCC-9 cells. (a) Tumor volume in the sh-DCST1-AS1 and sh-NC group. (b) Tumor weight in the sh-DCST1-AS1 and sh-NC group. (c, d) Ki-67 staining in the tumor tissues. ^∗^*P* < 0.05.

Then, we confirmed the effects of DCST1-AS1 on OSCC in vivo. Dorsal flanks of nude mice were injected with 1 × 10^7^ sh-DCST1-AS1- or sh-NC-transfected SCC-9 cells. Tumor volume in sh-DCST1-AS1 was repressed by DCST1-AS1 shRNA in a time-dependent manner (Figure 2(a)). Tumor weight in the sh-DCST1-AS1 group was greatly reduced (Figure 2(b)). In Figures 2(c) and 2(d), the Ki-67-positive cell ratio was significantly repressed by sh-DCST1-AS1 in the tumor tissues.

### 3.3. Silencing of DCST1-AS1 Repressed M2-Like Polarization of Macrophages

**Figure 3:**
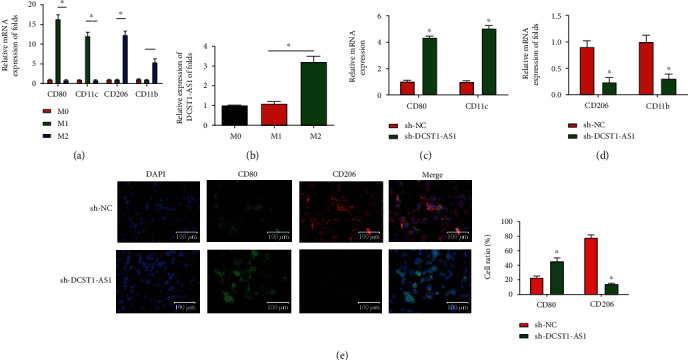
Silencing of DCST1-AS1 inhibited M2-like polarization of macrophages. (a) qRT-PCR was used to detect the expression of M1 markers and M2 markers. (b) The expression of DCST1-AS1 was increased in M2 macrophages. (c) Expression of M1 markers (CD80 and CD11c). (d) Expression of M2 markers (CD206 and CD11b). (e) Immunofluorescence of CD80 and CD206 in THP-1-differentiated macrophages transfected with sh-DCST1-AS1 or sh-NC. Scale bar, 100 *μ*m. The bar graphs indicated the quantification of the fluorescent puncta data. ^∗^*P* < 0.05.

Tumor-associated macrophages are considered to exhibit an M2-like phenotype in the tumor microenvironment. Then, to investigate whether DCST1-AS1 induced M2 polarization, unpolarized macrophages, LPS/INF-*γ*-induced M1 macrophages, and IL-4/IL-13-induced M2 macrophages were obtained. Then, we tested the expression of DCST1-AS1, M1 markers, and M2 markers. The expression levels of M1-associated genes (CD80 and CD11c) were elevated in M1 macrophages; meanwhile, M2-associated genes, including CD206 and CD11b, were also enhanced in M2 macrophages as confirmed in Figure 3(a). In addition, in Figure 3(b), lncRNA DCST1-AS1 expression was greatly increased in M2 macrophages compared to M1 macrophages. After PMA incubation for 24 h, THP-1 cells were transfected with sh-NC or DCST1-AS1 shRNA. The results indicated that M1 markers were significantly enhanced (Figure 3(c)), while M2 markers were downregulated in the DCST1-AS1 shRNA group (Figure 3(d)). Additionally, as shown in Figure 3(e), immunofluorescence of CD80 was increased while CD206 was decreased in THP-1-differentiated macrophages transfected with sh-DCST1-AS1.

### 3.4. Decrease in DCST1-AS1 Inactivated the NF-*κ*B Signaling Pathway

**Figure 4:**
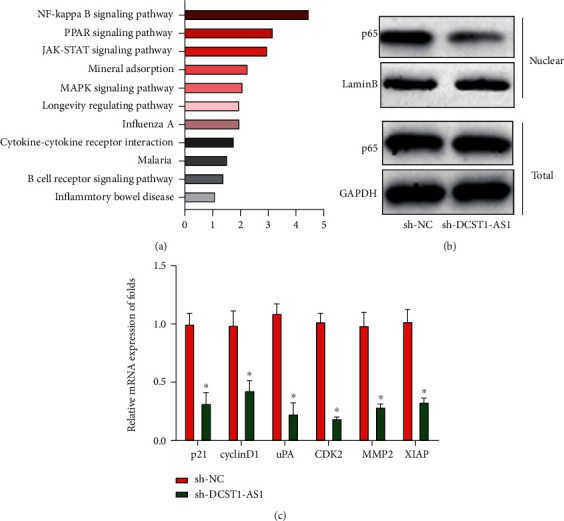
Loss of DCST1-AS1 inactivated the NF-*κ*B signaling pathway. (a) Pathway enrichment of the differently expressed genes regulated by DCST1-AS1 shRNA. (b) The expression level of total p65 and nuclear p65 in the THP-1-differentiated macrophages. (c) The expression level of downstream genes of the NF-*κ*B signaling pathway. ^∗^*P* < 0.05.

To further evaluate the function of DCST1-AS1 in M2-like polarization of macrophages, we performed RNA-seq in THP-1-differentiated macrophages transfected with DCST1-AS1 shRNA or sh-NC. Pathway enrichment of the differently expressed genes regulated by DCST1-AS1 shRNA was exhibited, and the NF-*κ*B signaling pathway was investigated as displayed in Figure 4(a). In addition, we observed that the expression level of nuclear p65 was obviously reduced in the THP-1-differentiated macrophages transfected with DCST1-AS1 shRNA (Figure 4(b)). For another, the expression level of downstream genes of the NF-*κ*B signaling pathway was significantly downregulated by loss of DCST1-AS1 in Figure 4(c).

### 3.5. Blocking the NF-*κ*B Pathway Inhibited the Progression of OSCC Cells and M2-Like Polarization of Macrophages Induced by DCST1-AS1

**Figure 5:**
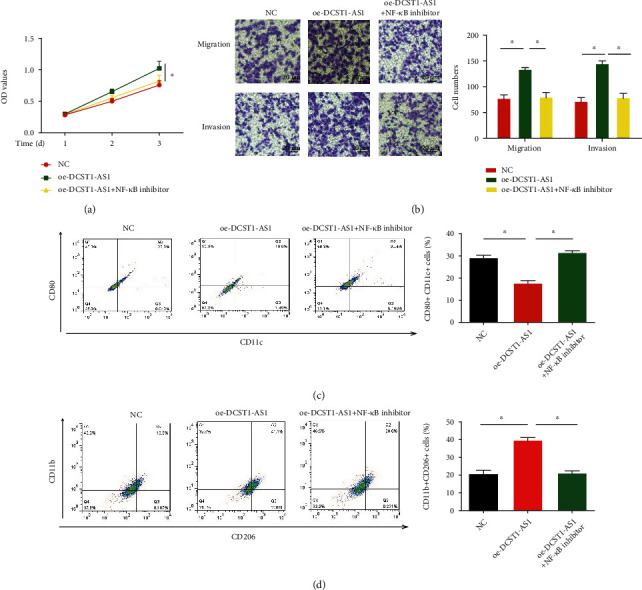
Blocking the NF-*κ*B pathway inhibited the progression of OSCC cells and M2-like polarization of macrophages triggered by overexpression of DCST1-AS1. SCC-9 cells transfected with oe-DCST1-AS1 were treated with or without the NF-*κ*B antagonist. (a) SCC-9 cell viability evaluated using the CCK-8 assay. (b) Transwell assay was used to assess SCC-9 cell migration and invasion capacity. THP-1-differentiated macrophages transfected with oe-DCST1-AS1 were incubated with or without the NF-*κ*B antagonist. (c, d) Flow cytometry analysis of M1 markers (CD11c and CD80) and M2 markers (CD11b and CD206). ^∗^*P* < 0.05.

Moreover, SCC-9 cells transfected with oe-DCST1-AS1 were incubated with or without the NF-*κ*B inhibitor. As shown in Figure 5(a), SCC-9 cell viability was induced by DCST1-AS1 overexpression, which was reversed by the NF-*κ*B inhibitor BAY 11-7802. In Figure 5(b), OSCC cell migration and invasion were reduced by BAY 11-7802. Furthermore, flow cytometry analysis was carried out, and we found that M1 markers (CD11c and CD80) were upregulated while M2 markers (CD11b and CD206) were remarkably downregulated by the NF-*κ*B inhibitor as demonstrated in Figures 5(c) and 5(d).

## 4. Discussion

The therapeutic methods to treat OSCC patients are complex, and their clinical outcome is not satisfactory. It is important to understand the molecular mechanisms of OSCC progression. In recent years, aberrant lncRNAs are reported in OSCC, and they can play important roles in OSCC progression [24–26]. Therefore, many studies have concentrated on the functions of lncRNAs in OSCC. Advances in the pathogenesis of OSCC are in urgent need to develop novel treatments. In our current study, we determined that the uncharacterized DCST1-AS1 was significantly increased in OSCC cells. We displayed that lncRNA DCST1-AS1 was closely associated with macrophage polarization and OSCC cancer progression. We found that DCST1-AS1 activated the NF-*κ*B pathway to promote OSCC development and M2 macrophage polarization.

The functionality of DCST1-AS1 is widely studied in various cancers. For example, lncRNA DCST1-AS1 induces CDK6 in cervical squamous cell carcinoma via sponging miR-107 [27]. DCST1-AS1 acts as a ceRNA to regulate expression of FAIM2 through sponging miR-1254 in hepatocellular carcinoma [28]. In addition, lncRNA DCST1-AS1 was increased in endometrial carcinoma via sponging miR-92a-3p and inducing Notch1 [29]. We found that DCST1-AS1 was increased in OSCC. Besides these, loss of DCST1-AS1 resulted in decreased proliferation, migration, and invasion of CSCC cells. Hence, we found that DCST1-AS1 is an oncogenic lncRNA in OSCC. In our future study, we would like to test whether the developed DCST1-AS1 inhibitor could repress OSCC progression in vivo.

Within tumor microenvironment, tumor-associated macrophages are major inflammatory cells [30, 31]. In general, tumor-associated macrophages can exhibit M2 phenotypes and induce tumor metastasis through releasing proteolytic enzymes and cytokines [32]. Tumor-associated macrophages predominantly polarize toward M2-like macrophages and contribute to the malignant tumor progression [33, 34]. Therefore, identifying the factors in M2 macrophage polarization is critical to inhibit tumor-associated macrophage-mediated cancer progression. For example, RACK1 induces OSCC progression by increasing the M2/M1 macrophage ratio [35]. The relationship between tumor-associated macrophages and OSCC is controversial. We indicated that DCST1-AS1 promoted M2 macrophage polarization.

As reported, inflammation is a major drive for recruiting immune cells such as macrophages and it is closely linked to tumorigenesis. NF-*κ*B is one of the major inflammatory regulators, and accumulating evidence indicates an aberrant activation of NF-*κ*B signaling and cancer progression [36–38]. NF-*κ*B is reported to modulate the expression of downstream inflammatory cytokines [39, 40]. More importantly, tumor-associated macrophages have been shown to produce interleukins to promote tumorigenesis via NF-*κ*B-mediated signaling [41, 42]. Based on these data, the blocking of NF-*κ*B signaling could represent a therapeutic target for treating OSCC [35, 43]. Our findings suggested that lncRNA DCST1-AS activates NF-*κ*B, which leads to OSCC progression and M2 macrophage polarization. Blocking NF-*κ*B signaling repressed the progression of OSCC cells and M2-like polarization of macrophages induced by DCST1-AS1.

In summary, we revealed that DCST1-AS1 promoted OSCC progression and M2 macrophage polarization via activating NF-*κ*B signaling. Our study suggested that DCST1-AS1 might be used as a valuable prognostic indicator and provided promising targets for OSCC patients.

## Figures and Tables

**Table 1:** Primers for real-time PCR.

Genes	Forward (5′-3′)	Reverse (5′-3′)
GAPDH	ATGGGTGTGAACCATGAGAA	GTGCTAAGCAGTTGGTGGTG
DCST1-AS1	CCACTCACCAGCTTCTTC	CTTCTGCTATGTCTCACCC
p21	ATGTAAGCTTATGTCAGAACCGGCTGGG	AAGEGAATTCTTAGGGCTTCCTCTTGGAG
XIAP	GCGAATTCGCCACCATGACTTTTAACAGTTTTGAAG	GCAGCCTCGAGGCAGACATAAAAATTTTTTGCTTG
CD80	ACGTCAAAGCAGTAGTCAAGG	GGAGGCCCTATGGAAAGTTAC
CD206	ATCCACTCTATCCACCTTCA	TGCTTGTTCATATCTGTCTTCA
CD11c	CAGGCATCATCCGCTAT	GGTCTCCGTACCCTCAAT
CD11b	ACTGGTGAAGCCAATAACGCA	TCCGTGATGACAACTAGGATCTT
Cyclin D1	CCCTCGGTGGGTCCTACTTCAA	TGGCATTTTGGAGAGGAAGT
uPA	AAATGCTGTGTGCTGCTGAC	AGGCCATTCTCTTCCTTGGT
CDK2	GCGAATTCCCCAGCCCTAATCTCA	GCCTCGAGAACCCTCTTCAGCAATAA
MMP2	CAAGTGGGACAAGAACCAGA	CCAAAGTTGATCATGTC

## Data Availability

The data used to support the findings of this study are included within the article.

## References

[1] Lala M., Chirovsky D., Cheng J. D., Mayawala K. (2018). Clinical outcomes with therapies for previously treated recurrent/metastatic head-and-neck squamous cell carcinoma (R/M HNSCC): a systematic literature review. *Oral Oncology*.

[2] Chi A. C., Day T. A., Neville B. W. (2015). Oral cavity and oropharyngeal squamous cell carcinoma--an update. *CA: a Cancer Journal for Clinicians*.

[3] Sasahira T., Kirita T. (2018). Hallmarks of cancer-related newly prognostic factors of oral squamous cell carcinoma. *International Journal of Molecular Sciences*.

[4] Iguchi K., Kawabata M., Arashima Y., Kubo N., Yoshida M., Kawano K. (1991). Studies on 15 seropositive cases to Lyme disease using immunoperoxidase test in Japan. *Kansenshōgaku Zasshi*.

[5] Wang N., Liang H., Zen K. (2014). Molecular mechanisms that influence the macrophage M1-M2 polarization balance. *Frontiers in Immunology*.

[6] Kitamura T., Qian B. Z., Pollard J. W. (2015). Immune cell promotion of metastasis. *Nature Reviews. Immunology*.

[7] Solinas G., Schiarea S., Liguori M. (2010). Tumor-conditioned macrophages secrete migration-stimulating factor: a new marker for M2-polarization, influencing tumor cell motility. *Journal of Immunology*.

[8] St Laurent G., Wahlestedt C., Kapranov P. (2015). The landscape of long noncoding RNA classification. *Trends in Genetics*.

[9] Charles Richard J. L., Eichhorn P. J. A. (2018). Platforms for investigating lncRNA functions. *SLAS TECHNOLOGY*.

[10] Kopp F., Mendell J. T. (2018). Functional classification and experimental dissection of long noncoding RNAs. *Cell*.

[11] Rinn J. L., Chang H. Y. (2012). Genome regulation by long noncoding RNAs. *Annual Review of Biochemistry*.

[12] Quinn J. J., Chang H. Y. (2016). Unique features of long non-coding RNA biogenesis and function. *Nature Reviews. Genetics*.

[13] Batista P. J., Chang H. Y. (2013). Long noncoding RNAs: cellular address codes in development and disease. *Cell*.

[14] Sun M., Kraus W. L. (2015). From discovery to function: the expanding roles of long noncoding RNAs in physiology and disease. *Endocrine Reviews*.

[15] Gao P., Wei G. H. (2017). Genomic insight into the role of lncRNA in cancer susceptibility. *International Journal of Molecular Sciences*.

[16] Cao H. L., Liu Z. J., Huang P. L., Yue Y. L., Xi J. N. (2019). lncRNA-RMRP promotes proliferation, migration and invasion of bladder cancer via miR-206. *European Review for Medical and Pharmacological Sciences*.

[17] Wang T., Tang X., Liu Y. (2019). lncRNA-ATB promotes apoptosis of non-small cell lung cancer cells through MiR-200a/*β*-Catenin. *Journal of BUON*.

[18] Luan J., Jiao C., Kong W. (2018). circHLA-C plays an important role in lupus nephritis by sponging miR-150. *Mol Ther Nucleic Acids.*.

[19] Niu X., Yang B., Liu F., Fang Q. (2020). lncRNA HOXA11-AS promotes OSCC progression by sponging miR-98-5p to upregulate YBX2 expression. *Biomedicine & Pharmacotherapy*.

[20] Zhang C., Bao C., Zhang X., Lin X., Pan D., Chen Y. (2019). Knockdown of lncRNA LEF1-AS1 inhibited the progression of oral squamous cell carcinoma (OSCC) via Hippo signaling pathway. *Cancer Biology & Therapy*.

[21] Yu L., Huo L., Shao X., Zhao J. (2020). lncRNA SNHG5 promotes cell proliferation, migration and invasion in oral squamous cell carcinoma by sponging miR-655-3p/FZD4 axis. *Oncology Letters*.

[22] Tang L., Chen Y., Chen H. (2020). DCST1-AS1 promotes TGF-*β*-induced epithelial-mesenchymal transition and enhances chemoresistance in triple-negative breast cancer cells via ANXA1. *Frontiers in Oncology*.

[23] Li J., Zhai D. S., Huang Q., Chen H. L., Zhang Z., Tan Q. F. (2019). lncRNA DCST1-AS1 accelerates the proliferation, metastasis and autophagy of hepatocellular carcinoma cell by AKT/mTOR signaling pathways. *European Review for Medical and Pharmacological Sciences*.

[24] Momen-Heravi F., Bala S. (2018). Emerging role of non-coding RNA in oral cancer. *Cellular Signalling*.

[25] Zhang L., Meng X., Zhu X. W. (2019). Long non-coding RNAs in oral squamous cell carcinoma: biologic function, mechanisms and clinical implications. *Molecular Cancer*.

[26] Feng L., Chen W. T., Qiu W. L. (2019). Long non-coding RNAs associated with oral squamous cell carcinoma. *European Review for Medical and Pharmacological Sciences*.

[27] Zhou Z., Xia N. (2020). lncRNA DCST1-AS1 sponges miR-107 to upregulate CDK6 in cervical squamous cell carcinoma. *Cancer Management and Research*.

[28] Chen B., Li Y., Liu Y., Xu Z. (2019). circLRP6 regulates high glucose-induced proliferation, oxidative stress, ECM accumulation, and inflammation in mesangial cells. *Journal of Cellular Physiology*.

[29] Ke J., Shen Z., Hu W. (2020). lncRNA DCST1-AS1 was upregulated in endometrial carcinoma and may sponge miR-92a-3p to upregulate Notch1. *Cancer Management and Research*.

[30] Farajzadeh Valilou S., Keshavarz-Fathi M., Silvestris N., Argentiero A., Rezaei N. (2018). The role of inflammatory cytokines and tumor associated macrophages (TAMs) in microenvironment of pancreatic cancer. *Cytokine & Growth Factor Reviews*.

[31] Cao J., Liu J., Xu R., Zhu X., Zhao X., Qian B. Z. (2017). Prognostic role of tumour-associated macrophages and macrophage scavenger receptor 1 in prostate cancer: a systematic review and meta-analysis. *Oncotarget*.

[32] Quatromoni J. G., Eruslanov E. (2012). Tumor-associated macrophages: function, phenotype, and link to prognosis in human lung cancer. *American Journal of Translational Research*.

[33] Najafi M., Hashemi Goradel N., Farhood B. (2019). Macrophage polarity in cancer: a review. *Journal of Cellular Biochemistry*.

[34] Mantovani A., Sozzani S., Locati M., Allavena P., Sica A. (2002). Macrophage polarization: tumor-associated macrophages as a paradigm for polarized M2 mononuclear phagocytes. *Trends in Immunology*.

[35] Dan H., Liu S., Liu J. (2020). RACK1 promotes cancer progression by increasing the M2/M1 macrophage ratio via the NF-*κ*B pathway in oral squamous cell carcinoma. *Molecular Oncology*.

[36] Dolcet X., Llobet D., Pallares J., Matias-Guiu X. (2005). NF-kB in development and progression of human cancer. *Virchows Archiv*.

[37] Hoesel B., Schmid J. A. (2013). The complexity of NF-*κ*B signaling in inflammation and cancer. *Molecular Cancer*.

[38] DiDonato J. A., Mercurio F., Karin M. (2012). NF-*κ*B and the link between inflammation and cancer. *Immunological Reviews*.

[39] Baldwin A. S. (1996). The NF-*κ*B and I*κ*B proteins: new discoveries and insights. *Annual Review of Immunology*.

[40] Pahl H. L. (1999). Activators and target genes of Rel/NF-*κ*B transcription factors. *Oncogene*.

[41] Lawrence T. (2011). Macrophages and NF-*κ*B in cancer. *Current Topics in Microbiology and Immunology*.

[42] Mancino A., Lawrence T. (2010). Nuclear factor-kappaB and tumor-associated macrophages. *Clinical Cancer Research*.

[43] Lee C. H., Liu S. Y., Chou K. C. (2014). Tumor-associated macrophages promote oral cancer progression through activation of the Axl signaling pathway. *Annals of Surgical Oncology*.

